# *Staphylococcus lugdunensis* Cultured from the Amniotic Fluid at Caesarean Section

**DOI:** 10.1371/journal.pone.0056373

**Published:** 2013-02-07

**Authors:** Zbigniew Marchocki, Kevin Collins, Eimear Lehane, Paddy O' Reilly, Keelin O'Donoghue

**Affiliations:** 1 Anu Research Centre, Department of Obstetrics and Gynaecology, University College Cork, Cork University Maternity Hospital, Cork, Ireland; 2 Department of Microbiology, University College Cork, Cork, Ireland; University of Illinois at Chicago College of Medicine, United States of America

## Abstract

*Staphylococcus lugdunensis* is a virulent coagulase-negative staphylococcus. It behaves like and can be mistaken in culture for *Staphylococcus aureus*. While originally thought to be a skin commensal rarely responsible for opportunistic infection, it was rapidly established as a significant human pathogen. It has been mainly associated with native and prosthetic valve endocarditis, osteomyelitis, and skin and soft tissue cellulitis, but has also been reported as a cause of fasciitis as well as peritonitis. *Staphylococcus lugdunensis* has been reported as a cause of endometritis but has not been previously isolated from amniotic fluid. Here, amniotic fluid samples were collected in the course of a larger study on amniotic fluid bacteriology, with prior ethical approval and informed patient consent. Amniotic fluid was obtained at Caesarean Section by direct needle aspiration from the intact amnion. Analysis with *Staphylococcal* API test kits led to identification of *Staphylococcus lugdunensis* in two cases. The clinical significance of the finding in these reported cases is undetermined. *Staphylococcus lugdunensis* has been shown to be a cause of serious and potentially fatal morbidities, but this is the first report of its culture from amniotic fluid. As caesarean delivery is accepted as the single most important factor associated with post-partum infectious complications in both mother and neonate, the identification of this pathogen is a new concern.

## Introduction

*Staphylococcus lugdunensis* is a gram-positive, catalase-positive, coagulase-negative *staphylococcus*. The organism was initially isolated and described in 1988 by Freney *et al*. [1]. It was first accepted as a skin commensal or occasionally opportunistic pathogen, and found to colonise mostly the groin and perineal regions [2]. However evidence published over the last decade confirm its life threatening potential both in immunosuppressed and immune-competent individuals. *S. lugdunensis* has now been shown to be an aetiological factor in cardiovascular infections, including native and prosthetic valve endocarditis, bloodstream infection (sepsis or septic shock), toxic shock syndrome, acute oral infection, urinary tract infection, bone and joint infection (infective arthritis or osteomyelitis, as well as prosthetic joint infection), central nervous system infection (brain abscess and meningitis), peritonitis and ocular infection [3], [4]. It is also reported as a cause of painful and prolonged skin and soft tissue infections [4], [5].

*S. lugdunensis* has been previously described in isolated case reports of post-partum infection but has not been reported isolated from amniotic fluid [6], [7]. Caesarean section (CS) is an important risk factor for postpartum maternal infection [8], [9]. Here, we present two cases of pregnant women who underwent CS and were incidentally found to have amniotic fluid colonised with this potentially dangerous pathogen.

## Methods

Amniotic fluid (AF) samples were collected in the course of a larger study on amniotic fluid bacteriology. The study was approved by the Clinical Research Ethics Committee of the Cork Teaching Hospitals [ref ECM 4: 02/08/2011] and written informed consent was obtained from each participant prior to CS. In each case a different operator (both senior clinicians) in a different operating theatre performed the CS, and the deliveries were weeks apart. As per hospital policy at the time, routine intravenous antibiotic prophylaxis was administered after delivery of the infant at CS in both cases.

AF samples were obtained at the time of CS by direct syringe aspiration from the intact amnion and stored at 4°C until processed. Samples were plated on Colombia Blood Agar. Subsequent Gram Stain, Catalase Test, Baird-Parker Selection Agar Plates, Rapid Agglutination Test and finally, analysis in *Staphylococcal* API test kits (bioMérieux, UK) led to identification of the *S. lugdunensis* species.

## Results

The first case was a 38-year-old multiparous white woman who underwent elective repeat caesarean section (CS) at 37 weeks and 6 days. She had an emergency CS in her first pregnancy for uterine rupture in the second stage of labour. This pregnancy course was uneventful and she had no medical co-morbidities. She was a non-smoker and was overweight (body mass index (BMI), 28). Elective surgery was carried out without any complications and a liveborn infant of normal birth weight was delivered. Mother and baby left hospital four days after CS, with no infectious post-operative complications reported.

The second case was a 30-year-old nulliparous white woman, who underwent induction of labour with prostaglandin E2 (PGE2) for post-maturity. Her pregnancy course was uneventful and her medical co-morbidities included well-controlled asthma. She smoked 20 cigarettes daily prior to pregnancy and had a BMI of 25. Membranes spontaneously ruptured 7 hours after administration of PGE2 after which she was commenced on a syntocinon infusion. She subsequently underwent an emergency CS for fetal distress- an abnormal fetal blood sample (pH 7.14) was obtained following non-reassuring features evident on cardiotocography. Emergency caesarean section was performed and amniotic fluid obtained through the bulging amnion. A liveborn infant of normal birth weight was delivered without complication. Again, mother and baby left hospital four days after CS, with no infectious post-operative complications reported.

In both cases clear amniotic fluid was obtained by direct needle aspiration from the intact amnion at CS. Bacterial colonisation was noted in both AF samples; 90 colony forming units per ml (CFU/ml) in the first sample, and 550 CFU/ml in the second under aerobic conditions, with few under anaerobic conditions. *S. lugdunensis* species was subsequently identified in both AF samples.

## Discussion

For many years, it was assumed that the amniotic fluid cavity prior to onset of labour was a sterile environment. Harris and Brown in 1927 investigated the presence of bacteria in amniotic fluid of women undergoing CS, to find that all women who were in labour for <6 hours had negative cultures, whereas those who were in labour for >6 hours had positive cultures [10]. Many reports have since confirmed that the amniotic cavity is not entirely sterile. Multiple organisms have been found using traditional bacterial culture methods and more recently, using advanced molecular techniques. Organisms identified include species of *Ureaplasma, Mycoplasma, Fusobacterium, Streptococcus, Staphylococcus, Bacteroides* and *Prevotella*.

Caesarean section (CS) is now a common surgical procedure, performed to deliver around 25% of all pregnancies. As CS has become safer and electively scheduled, functional and cosmetic aspects, as well as risks of hospital-acquired infection have gained increased importance. A CS delivery is the single most important factor associated with post-partum infection and carries a 5–20 fold increased risk of infection compared to vaginal delivery [9]. Endometritis, surgical site or urinary tract infections occur in about 8% of women who have a CS [8]. Case reports of early neonatal sepsis also suggest that the infection process is likely to be triggered in utero [11]. Risk factors associated with infection after CS include maternal obesity, prolonged operating time, poor surgical technique and emergency delivery [8], [9]. While the majority of surgical site infections are not life threatening they have important implications on the length of hospital stay, hospital costs and re-admission rates [9]. Around 80% of infections occur after discharge from hospital. With the increase in CS rates worldwide, it is anticipated that post CS infections will become an increasing health and economic burden [8], [9].

Here, we report two women in whom amniotic fluid was colonised with the gram-positive bacteria, *S. lugdunensis*, at the time of CS. This organism has been already shown to be a cause of serious and potentially fatal morbidities in other medical specialities, and has been reported as a cause of post-partum endometritis and pyomyoma [6], [7]. It has never been demonstrated at CS before now. CS is accepted as a major risk factor in post-partum infectious complications and the identification of this pathogen is a new concern. Further, the identification of *S. lugdunensis* can lead to confusion or misdiagnosis, as it can be easily mistaken for *Staphylococcus aureus* due to its production of the clumping factor that gives a positive result in slide coagulase or rapid latex agglutination tests [3].

Our findings could occur secondary to contamination, as *S. lugdunensis* is a skin commensal colonising the groin area [2]. However, we found the bacterium in samples taken by different senior operators on different days. Operators were trained in an aseptic technique of sampling, and neither sample was macroscopically or microscopically contaminated by blood. We could not facilitate two separate samples of AF being taken and analysed independently, given the clinical necessity of CS. In each case, the CS was performed for a different indication, one being emergency and the other elective (although the previous serious complication in the elective CS is noted and could be relevant). In both cases, standard aseptic techniques were observed in hand decontamination and antiseptic skin preparation prior to skin incision at CS. No infectious post-operative or neonatal complications were observed in either case before discharge, but neither was followed up in the hospital. Finally, procedures were standardised in a large accredited and internationally-recognised research laboratory. The clinical significance of the findings of *S. lugdunensis* in these reported cases therefore remains undetermined.

## Conclusions

This is the first case report of *S. lugdunensis* cultured from amniotic fluid during caesarean section. CS delivery is the single most important factor associated with post-partum infectious complications, and the identification of this pathogen is a new concern. Further studies are needed to examine the prevalence of *S. lugdunensis* colonisation in amniotic fluid and to establish if there is an association with post-operative maternal or neonatal infectious morbidity, particularly after emergency CS.
